# Relationships between moderate vigorous physical activity, motor- and health-related fitness and motor skills in children

**DOI:** 10.4102/phcfm.v16i1.4258

**Published:** 2024-05-20

**Authors:** Carli Gericke, Anita E. Pienaar, Barry Gerber, Makama A. Monyeki

**Affiliations:** 1 Physical Activity, Sport and Recreation (PhASRec), Faculty of Health Science, North-West University, Potchefstroom, South Africa; 2 School of Human Movement Sciences, Faculty of Health Science, North-West University, Potchefstroom, South Africa

**Keywords:** children, health-related physical fitness, motor skills, physical activity, socioeconomic status

## Abstract

**Background:**

Childhood is an important transitional period for the development of healthy physical activity (PA) behaviours, so it is important to understand its impact on a healthy lifestyle.

**Aim:**

This study aimed to determine the influences of sex, socioeconomic status (SES) and body composition (BC) on the relationships between PA, motor skills, motor- and health-related physical fitness in 5–8-year-olds.

**Setting:**

Participants were a subsample consisting of 299 children (150 boys, 149 girls, mean age 6.83 ± 0.96 years) from the Exercise, Arterial Modulation and Nutrition in Youth South Africa study (ExAMIN Youth SA).

**Methods:**

Anthropometric measures, health-related physical fitness (HRPF), motor-related physical fitness (MRPF), objectively measured PA and demographic information were determined.

**Results:**

Only 66% achieved the recommended 60 min of daily moderate vigorous physical activity (MVPA) with 19% classified as having unhealthy body composition (11% overweight, 8% obese). Fat-free mass and SES revealed small-to-moderate influences on the relationship between MVPA, standing broad jump (SBJ; *r* = 0.32), predicted VO_2_max (*r* = 0.28) and beep levels (*r* = 0.22). For MRPF, the quality of running (*r* = 0.12) and balancing were associated with MVPA. Adjusting for sex, BC and SES in the relationship between PA with HRPF and MRPF, reductions in most correlations were observed.

**Conclusion:**

Moderate vigorous physical activity levels were positively associated with HRPF, MRPF and some motor skills in 5–8-year-olds. Socioeconomic status (lower parental income, employment and education negatively influenced the association between MVPA and fitness [beeps, SBJ, $\dot{\text{V}}$O_2_max]).

**Contribution:**

This study provides knowledge with regard to the use of accelerometer for baseline data for PA, MRPF, HRPF as well as motor skills in South African children.

## Introduction

Physical activity (PA), physical fitness (PF) and motor competence (MC) are worldwide declining, indicating global health problems in children.^1,2,3,4,5,6,7^ In this respect, physical inactivity is globally considered the fourth most important risk factor for mortality.^1^ In addition, the adverse effects of inadequate PA on current and long-term health outcomes^1,8^ are associated with obesity and increased weight in children and adults^9^ and are the basis for research.

Physical activity is described as any movement of the body performed by skeletal muscles and generates energy expenditure.^5^ Studies using self-reported PA measurements reported a decline in PA by about 10 years of age,^10^ while recent objectively measured PA results show that these trends occurred at 7 years of age.^11^ More specifically, PF contributes to PA and is described as a condition that allows and supports daily performance, work, learning, recreation and movement of PA. Furthermore, PF can be divided into health-related (HRPF) and motor-related physical fitness (MRPF),^12,13^ which are found to be related with MC in early childhood.^12,13^ Motor competency therefore is described an umbrella concept for gross goal-oriented movements involving large muscles or entire body activities (such as running, jumping and balance).^7^ Physical activity plays an important role in the achievement or improvement of MC.^14^ In this regard, MC is considered a key enabler of children’s PA^2^ and is associated with PA throughout the lifespan^4,15^ as it promotes participation in PA that contributes to PF during early, mid and late childhood.^15^

Importantly, the WHO^1^ reports that less than 20% of children and 80% of adolescents worldwide achieve the recommended 60 min daily of moderate vigorous physical activity (MVPA). While the Global Matrix (2018) reported that the levels of PA in children aged 11 and 14 years are 88% and 66%, respectively achieved the recommended 60 min daily.^16^ From South African perspective, the 2016 Healthy Active Kids South Africa Report Card (HAKSA) revealed that only 50% of SA children meet the daily requirements of 60 min of MVPA, consisting mostly of aerobic PA. However, the 2018 HAKSA Report Card does not provide any new evidence of an increase in PA.^17^ Furthermore, researchers reported a 7% decrease in PA among South African adolescents in a year, while only 50% of South African children aged 9–11 years participated in team sports.^18^ The PA levels of preschool and grade R children living in low-income rural settings in South Africa have shown that preschool children are significantly more active than grade R children and spent more time in light-intensity PA (8.6% vs. 2.7%) and MVPA (25.4% vs. 15.3%).^16^

In addition, strong reciprocal relationship was also reported between PA, PF and MC among young children.^19^ A study by Haga^20^ found that MC components such as manual dexterity, ball skills and balance are significant predictors of PA and PF levels, explaining the association between MC and PF.^20^ It was reported that physically fit children tended to participate in PA and maintain their PA behaviour in adolescence. Such behaviour is found to be associated with improved PF (cardiorespiratory and muscular fitness), cardiometabolic health (blood pressure, dyslipidaemia, glucose and insulin resistance), bone health, cognitive outcomes (academic performance, executive functioning), mental health (reduced symptoms of depression) and reduced obesity.^1,4,15^ Physical fitness is also an integrated measurement of most body functions involved in performing daily PA and is considered an important indicator of health in childhood.^21^

The achievement of MC, PA and PF depends on many factors that affect these health enablers in children,^15^ including but not limited to age, sex, body composition (BC), living conditions and socioeconomic status (SES).^4,5^ The sex differences in PA can also be explained by environmental influences (school and community-based PA, free play and parental support), biological factors or their interaction.^22^ Age is also an important correlate of PF, as age-related growth, maturation and development show significant correlations with PF.^4,15^

In a study performed in the North West province of South Africa, BC measures and MC were associated with poor gross motor skills, which has being linked with overweight and obesity from early childhood to later childhood.^23^ Higher PA levels in early childhood are associated not only with favourable measures of BC but also with positive psychosocial, gross motor skill development and physical health outcomes.^23^ The interaction is reported between the child’s body movement and living conditions that can influence changes in motor development and MC.^24^ Factors such as parental education, occupation, household income, rural-urban differences and migration backgrounds contribute to SES.^25^ Studies have shown that changes in cardiorespiratory fitness (CRF), lower limb strength, balance, endurance sprint velocity and flexibility among 6–11 year-old children with educated parents are linked to increased participation in children’s sports and leisure activities, as well as greater understanding of the beneficial health effects of regular PA.^3,26^

International studies conducted on children from Italian, Polish, Austrian, Cypriot, Portuguese, Spanish and Slovakian have indirectly confirmed that children living in urban and rural communities differ not only in the PA (higher PA scores of children living in urban areas) but also in fitness status and anthropometric or physical indices.^27,28^ Children living in Australian rural areas having better gross motor skills than South African children living in low- and high-income urban areas^29,30,31^ also confirm a unique country-specific influence. The physical demand of indoor and outdoor activities of children,^5^ and the encouragement of parents to participate in activities play a critical role in organising of opportunities.^29^ Thus, in this regard, SES, ethnicity and cultural factors may also contribute to sedentary behaviours, which in turn influence children’s PF and MC status.^32,33,34^

Most of the published studies were not designed to examine the effect of various sociodemographic factors (age, gender, race and ethnicity, SES) that may have an influence on the health effects of PA. However, such information is important to provide more specific public health recommendations on the role of PA in the current and future health of young children and to reduce health disparities in the more vulnerable populations. This leaves a gap in the understanding of the multivariate association of factors such as sex, SES and BC on the well-established relationship between PA, PF and MC in older age groups but not in the age group between 5- and 8-year-old children living in South Africa.

The aim of this study was therefore to determine the multivariate associations of sex, SES (more specifically the role of parental education, employment status and income) and BC on relationships between objectively measured PA, specifically MVPA levels. These results will not only shed light on the beneficial effects of achieving recommended PA levels during childhood on the prevalence of obesity and overweight but also on associations with objectively measured PA, PF and MC that can be taken into consideration as strategies for further improvements of healthy behaviour.

## Research methods and design

### Study design

We followed a cross-sectional research design with available data from the Exercise, Arterial Modulation and Nutrition in Youth South Africa study (ExAMIN Youth) and the BC using Isotope Techniques (BC–IT) studies. The ExAMIN Youth Study is an analytical, multidisciplinary, observational cohort study designed to explore the interaction between BC, motor- (MRPF) and health-related physical fitness (HRPF) and PA and salivary biomarkers. The participants involved in the larger study consisted of approximately 1100 children (aged 5–8 years) attending 10 public primary schools in the North West province (6 schools in Potchefstroom and four schools in Klerksdorp area) of South Africa. The larger BC–IT study examined the relationships between the more complex measurements (using the methods of stable isotope and bioelectrical impedance analysis [BIA]) and the more indirect measurements of BC (using anthropometric variables). In addition, the larger study was conducted to determine the objective and subjective measures of PA among the children between the ages 6 and 8 in South African and their relationship with other health-related determinants. The complete methodology of the study can be found elsewhere^35^ and is briefly described next.

### Study population and sampling

The data of 299 children between the ages of five and eight (150 boys and 149 girls) are part of this study. In this study, children attending various primary schools in the Tlokwe Local Municipality of the North West province were included with complete data on ActiGraph and bioelectrical impedance (BIA) data were included. Briefly, children were excluded if no permission from parents was obtained if the child did not want to participate and if the child was ill on the day of the measurements. The average age of this subsample was 6.83 ± 0.96 years (5 years [*n* = 27], 6 years [*n* = 86], 7 years [*n* = 95] and 8 years [*n* = 91]). The Generalised Linear Model for Analysis of Variance was used to calculate the statistical power for the appropriate sampling size for a power of 0.80 and alpha level of 0.05 and a confidence interval (CI) of 95%.

### Data collection

#### General health and demographics questionnaire

Sociodemographic information about personal information and family (i.e. education of the parents, employment, type of dwelling, household amenities and marital status), lifestyle behaviours and health, were collected using a standard general health questionnaire. Parents or legal guardians assisted in the completion of the demographic questions.

#### Anthropometric measurements

All anthropometric measurements (e.g. height in centimetres [cm], weight in kilogram [kg], skinfolds in millimetres [mm]) were performed following International Society for the Advancement of Kinanthropometry (ISAK) standards,^36^ conducted by a qualified anthropometrist (i.e. a person who completed Level 1 can demonstrate adequate precision in four base measures, eight skinfolds, six girths and three breadths and has a basic understanding of the theory of anthropometric applications). Measurements were taken in separate rooms for the boys and girls in order to ensure privacy. A stadiometer (Seca 213, Holstein Limited, Crosswell, Crymych, UK) was used to measure height to the nearest 0.1 cm, where participants were required to stand barefoot with an upright posture and their head in the Frankfort plane.^36^ Weight was measured to the nearest 0.1 kg with a digital scale (Seca 813, Beurer Ps07 Electronic Scale, Ulm, Germany), while the participants wore minimal clothing without shoes. The waist circumference was measured at the narrowest point of the abdomen between the lower costal (10th) rib and hip using a metal tape (Lufkin, Cooper Industries, USA) to the nearest 0.1 cm. The body mass index (BMI) was calculated as weight divided by height squared (kg/m^2^) and standardised BMI values (z-scores) were calculated relative to World Health Organization (WHO) reference data. The WHO BMI z-score categories used were underweight: –2 standard deviation (s.d.) from the mean; normal weight: –2 s.d. to +1 s.d.; overweight: +1 to +2 s.d. and obesity: more than +2 s.d..^37^

#### Bioelectrical impedance analysis

The BIA measuring tool (BodyStat 1500^®^MDD) was used to determine the body fat percentage (BF%), fat mass (FM, kilogram), fat-free mass (FFM in kilograms) and total body water (TBW in liter [L]). The BIA procedures that were used were published previously^19,38^ and are also described in the BC–IT study^39^ in which the sub-study was based on the baseline data of the BC-IT study. In brief, the BC was assessed using BIA Bodystat^®^ with a measurement frequency of 50 kHz. Measurements of height, sex and age were entered manually, while body mass was recorded automatically using 0.5 kg to adjust for clothing weight in all subjects. The Bodystat1500^®^ software inbuilt prediction equations are used to produce an output specifying TBW, FFM (using a hydration factor of 0.73, independent of age), body fat (BF) and percentage body fat (%BF) as well as impedance, resistance and reactance readings. For the classification of fatness (underfat = 2nd, overfat = 85th and obese = 95th percentiles), the McCarthy^39^ was applied, which was developed in Caucasian children. The BIA prediction equations for TBW and FFM have been validated for use in Chinese, Lebanese, Malay, Filipino and Thai children aged 8 to 10 years (948 participants) across a wide BMI range (12.2 kg/m^2^ – 34.9 kg/m^2^).^40^ The details of the body status classifications in this sample are described in detail in our previous publication.^41^

#### Physical and motor fitness

The test protocols used include tests from different test batteries to assess HRPF characteristics (20 m shuttle run, predicted VO_2_max and leg strength). The 20 m shuttle run is a valid and recognised endurance test, which involves running through a 20 m distance back and forth,^42^ with a starting speed set at 8.0 km/h, and an increase of 0.5 km/h per minute followed by a sound beep on a stereo. When the participant does not cross the 20 m line at a 20 m-long beep sound or stops voluntarily, the test ends. This test protocol has been established to be reliable (*r* = 0.89) in children aged 6 to 16 years. The FitnessGram uses an equation that included field test scores, age, gender and BMI to determine aerobic capacity ($\dot{\text{V}}$O_2_max). The following equations were used to convert the attained beep levels to aerobic capacity: $\dot{\text{V}}$O_2_max = 45.619 + (0.353*Pacer laps) – (1.121*age).^41^ Motor-related physical fitness (10 m and 20 m speed and agility) and motor skills (process and product measurements of balancing, kicking and catching) were assessed by using selected tests from the Smartspeed, Körperkoordinationstest für Kinder (KTK)^43^ and the Test of Gross Motor Development-2 (TGMD-2)^44^ test batteries. Ten and 20-m speeds were assessed using the Smartspeed system (Fusion Sports, Summer Park, Australia) with a reliability of *r* = 0.9 in children aged 6 to 11 years.^45^

#### Test of Gross Motor Development-2

The TGMD-2 Protocol was used to assess the MC in each of the motor skills qualitatively. The TGMD-2 is a process-based test battery that assess the quality of performance in 12 gross motor skills.^44^ These 12 skills are divided into two sub-domains, namely object control skills (striking a stationary ball, stationary dribbling, catching, kicking, overhand throw and underhand roll) and locomotor skills (running, galloping, hopping, leaping and horizontal jump). Because of time constraints, the following four tests that represent two locomotor skills (running and jumping) and two object control skills (catching and kicking) were used. Jumping was representative of a qualitative measure of strength in HRPF, running was a qualitative measure of running speed in MRPF and catching and kicking were qualitative measures of motor skills. It is not uncommon for researchers to select only a few of these skills for their studies, while these four are also the most common ones that are often selected. These four locomotor and object control skills are also most relevant to typical South African sports and game activities. After a demonstration of the skill, two attempts of the skill were allowed and scored according to specific sub-criteria (0 = no mastery, 1 = mastery), after which the scores were added together. The same evaluators were used for each test item to ensure the reliability of the results. An overall validity coefficient of *r* = 0.89 is reported for the TGMD-2 with an internal consistency reliability coefficient (*r* = 0.85) for the locomotor and (*r* = 0.78) for the object control subdomains.

Competence in catching, kicking, running and horizontal jumping was also assessed quantitatively. Catching and kicking accuracy were additionally scored out of five attempts, also using the TGMD-2 protocol for distances between the tester and participant in catching and the distance to the kicking target of 1.5 cm wide). The distance jumped in the horizontal jumping test and the time to complete the 20 m speed test with the Smartspeed system (already described) were used as quantitative measures for speed and jumping, respectively. The horizontal jump was designed to measure explosive strength (muscular fitness) and was scored in centimetres.^44^ Two trials were given and the best score was recorded in centimetre. This test was performed on a non-slippery mat designed specifically for the horizontal jump.

#### Körperkoordinationstest für Kinder

The KTK test was developed in Germany to assess the motor coordination components in children and adolescents ranging from 5 to 14 years.^43^ The test consists of four tasks: (1) walking backward along a balance beam of decreasing width (6.0 cm, 4.5 cm and 3.0 cm, counting the number of successful steps); (2) two-legged jumping from side to side for 15 s; counting the number of jumps; (3) moving sideways on wooden boards for 20 s and (4) hopping for height (hopping over a foam obstacle with increasing height in consecutive steps of 5 cm. Only two of these test items were selected for use in this study to determine coordination, agility and muscular endurance (two-legged jumping) as well as balancing (balancing walking backward) to obtain a more comprehensive overview of the motor abilities of the participants. The KTK tests have test-retest reliability coefficients ranging between *r* = 0.80–0.96 and a reliability score of *r* = 0.97 for the raw scores. The intercorrelations between the four tests range from 0.60 to 0.81.^43^

#### Physical activity using ActiGraph Accelerometers

Physical activity and sedentary behaviour (SB) were assessed using the ActiGraph GT3X (model 7164; Fort Walton Beach, FL, USA), which is a solid-state triaxial accelerometer that is validated for use in children.^46^ All participants were fitted with the ActiGraphs devices that they had to wear around the waist just above the anterior superior iliac spine, according to the manufacturer’s instructions. Participants had to wear the ActiGraph for a minimum of 10 h/day for 7 consecutive days. However, the participants were allowed to remove the accelerometers during water-based activities or if they felt uncomfortable wearing the device during sleep. A daily log sheet had to be completed by the parents to indicate the time the Actigraph was worn and removed. Each participant also received an instruction manual on the proper usage of the accelerometer for additional guidance to the parent. The ActiLife software (Version 6.13.3) was used to extract and analyse the data. The PA data were expressed as average daily minutes spent in SB (< 99 counts per minute), light PA (LPA) ≥ 100 counts per minute, moderate PA (MPA) ≥ 2296 counts per minute and vigorous PA (VPA) ≥ 4012 counts per minute. Time in moderate-to-vigorous PA was calculated as the sum of moderate PA and vigorous PA. Participants who provided a minimum of 4 days of valid data, including 1 weekend day, were included in the analysis. Valid days were those days on which the accelerometer was worn for at least 600 min (10 h) per day. Consecutive zero counts for 20 min or more were as considered as no-wear time.

### Data analysis

The data were analysed by means of the Statistical Package for Social Sciences (SPSS v 28.0). The normality of the data was first determined using the Shapiro–Wilk test where deviations from normality were accepted at *p* < 0.05. Descriptive statistics, including frequencies, percentages, means and standard deviations, was used to characterise the research sample. The independent t-test was used to compare the differences in BC on PA and motor and HRPF between boys and girls aged 5 to 8-year-old. A Pearson chi-square was performed to determine group differences in the BIA classification. Spearman correlation coefficients (Rho) examined the relationships between PA and motor- and HRPF. For the interpretation of correlation coefficients measure of relationship strength (or effect size), the Cohen^47^ classifications were used: *r* = 0.1–0.29 is considered a weak correlation; *r* = 0.3–0.49 a moderate correlation and *r* = 0.5–1.0 a strong correlation. As influences were found for sex, SES and BC, a partial correlation analysis was performed to adjust for possible co-variants in this analysis. Statistical significance was accepted at *p* ≤ 0.05.

### Ethical considerations

The Health Research Ethical Committee of the Faculty of Health Science at the North-West University, Potchefstroom, SA, granted permission for the observational cohort/follow-up study, the ExAMIN Youth SA (NWU-00091-16-A1) and the cross-sectional BC-IT study (NWU-00025-17-S1) and this affiliated study (NWU-00457-20-A1). The parents or legal guardians had to give written consent, while all the participants had to provide verbal or written consent to participate in the study. Every third child on each class list was selected to participate in the study. Only those with signed parental informed consent forms who personally agreed to participate were finally included.

## Results

The participants included 299 individuals (150 boys and 149 girls) with a mean age of 6.83 (±0.96) years, with no significant (*p* = 0.75) age difference between the boys (6.85 ± 0.96) and the girls (6.82 ± 0.97). Table 1 describes the percentage contribution of the different aspects related to the three studied SES demographic variables of parents or guardians of the participants (i.e. education level, employment status and household income) of the group. In terms of the Education construct for SES a higher percentage (56%) of participants, respectively, had attended high school, primary school and Adult Based Education Training (ABET) education system with 2% with no education. In addition, the results show that a high percentage (32%) of the participant’s parents were unemployed. It was also found that 20% of the household income lives below the poverty line.

**TABLE 1:** Demographic characteristics (*n*, %) representing socioeconomic status of the group (*N* = 299).

Socioeconomic status	*n*	%
**Highest educational level of the parents or guardians**		
None	5	2
Primary school	11	4
High School	136	48
ABET (Adult-based education training)	11	4
College/University/other tertiary institution	119	42
**Employment status of parents or guardians**		
Unemployed	90	32
Employed	131	46
Self-employed	61	22
Total	282	-
**Household income of parents or guardians**		
Less than R1000	54	20
R1000 – R4999	45	17
R5000 – R9999	29	11
R10000 – R20000	44	17
More than R20000	91	35

*n*, number; %, percentage.

Table 2 shows the characteristics of the participants’ BC by the group and sex. There were no significant (*p* > 0.05) sex differences evident for height, weight, BMI, WC and TBW in litter for the group.

**TABLE 2:** Participants’ age, body composition and body mass index-based body fat characteristics per sex.

Variable	Total (*N* = 299)			Male (*n* = 150)			Female (*n* = 149)			Significance of sex differences (*p*)	Pearson chi-square for the sex differences
Body composition components	*n*	%	Mean ± s.d.	*n*	%	Mean ± s.d.	*n*	%	Mean ± s.d.		
Body height (cm)	-	-	122.58 ± 7.99	-	-	123.23 ± 7.94	-	-	121.91 ± 8.01	0.15	-
Body weight (kg)	-	-	24.46 ± 6.46	-	-	24.69 ± 6.57	-	-	24.24 ± 6.35	0.55	-
BMI (kg/m^2^)	-	-	16.08 ± 2.62	-	-	16.09 ± 2.60	-	-	16.08 ± 2.60	0.97	-
Waist circumference (cm)	-	-	54.89 ± 6.95	-	-	55.46 ± 6.73	-	-	54.31 ± 7.15	0.15	-
Body fat percentage (%)	-	-	23.45 ± 8.21	-	-	20.86 ± 7.60	-	-	26.05 ± 8.00	< 0.001	-
Fat mass† (kg)	-	-	6.73 ± 12.22	-	-	5.38 ± 3.37	-	-	6.73 ± 3.66	< 0.001	-
FFM† (kg)	-	-	18.51 ± 3.99	-	-	19.37 ± 4.19	-	-	17.64 ± 3.59	< 0.001	-
Total body water † (L)	-	-	14.74 ± 5.34	-	-	15.15 ± 4.75	-	-	14.32 ± 5.86	0.18	-
**BMI z-score based body fatness classification**											< 0.001
Underweight or thinness FM% < 18.5%	79	27	-	57	38	-	22	15	-	-	-
Normal FM% between 18.5% and 29.9%	162	54	-	77	52	-	85	57	-	-	-
Overweight FM% between 30 and 34.9	33	11	-	10	7	-	23	15	-	-	-
Obese FM% > 35%	24	8	-	5	3	-	19	13	-	-	-

s.d., standard deviation; *n*, number; BMI, body mass index; or BF%, body fat percentage; FFM, fat free mass/lean mass; FM, fat mass percentage; L, litre.

† , bioelectrical impedance.

Girls were significantly (*p* < 0.001) fatter (26.05 ± 8.00 BF%) compared with boys (20.86 ± 7.60 BF%) and had a significantly (*p* < 0.001) higher mean value for fat mass (6.73 kg ± 3.66 kg vs. 5.38 kg ± 3.37 kg). Boys had significantly (*p* < 0.001) higher mean values for FFM (19.37 kg ± 4.19 kg) compared with the girls (17.64 kg ± 3.59 kg). Out of 299, 79 (27%) of the children were underweight, 33 (11%) overweight and 24 (8%) obese. Boys were significantly more underweight (27%), while girls were, respectively, more overweight (15%) and obese (13%).

Table 3 presents the characteristics of MRPF (10 m and 20 m speed and agility), motor skills (balance, kicking and catching) and HRPF (beep, estimated $\dot{\text{V}}$O_2max_ and leg strength (standing broad jump [SBJ]) (means, standard deviations, minimum and maximum values) by the group, sex and the *p*-value of these differences. Significant sex differences (*p* < 0.001) were found in 10 m and 20 m speed run, SBJ, kicking, balance, catching, the beep and the $\dot{\text{V}}$O_2max_.

**TABLE 3:** Motor-related physical fitness and health-related physical fitness characteristics of the group and by sex.

Variable	Motor fitness and motor skills			Mean ± s.d.		*p*-value of sex differences
	Mean ± s.d.	Min	Max	Males	Females	
**Motor-related physical fitness**						
Speed 10 m (s)	2.41 ± 0.26	1.95	4.43	2.35 ± 0.21	2.47 ± 0.30	< 0.001*
Speed 20 m (s)	4.34 ± 0.45	2.26	6.59	4.23 ± 0.40	4.46 ± 0.48	< 0.001*
Agility (total)	42.18 ± 13.72	10.00	78.00	41.19 ± 12.68	43.18 ± 14.68	0.209
Balance quantitative (total)	35.50 ± 11.54	5.00	69.00	33.91 ± 11.60	37.11 ± 11.28	0.016*
Balance qualitative	2.34 ± 0.58	1.00	3.00	2.29 ± 0.57	2.39 ± 0.59	0.169
Catching quantitative (total)	3.40 ± 1.55	0.00	56.00	3.55 ± 1.55	3.25 ± 1.55	0.090
Catching qualitative (total)	5.19 ± 0.96	0.00	5.00	5.22 ± 1.55	5.17 ± 1.54	0.599
Kicking quantitative (total)	2.94 ± 1.33	0.00	5.00	3.20 ± 1.23	2.68 ± 1.39	< 0.001*
Kicking qualitative (total)	7.19 ± 1.25	1.00	8.00	7.62 ± 0.77	6.76 ± 1.48	< 0.001*
**HRPF**						
SBJ (cm)	113.08 ± 18.53	66.10	165.20	116.84 ± 18.74	109.30 ± 17.58	< 0.001*
SBJ qualitative (total)	6.01 ± 1.33	1.00	8.00	-	-	-
Beep (laps)	27.46 ± 12.97	4.00	78.00	29.82 ± 14.65	25.10 ± 10.57	0.002*
$\dot{\text{V}}$O_2max_	47.11 ± 4.30	37.85	64.40	47.95 ± 4.81	46.27 ± 3.54	< 0.001*

s.d., standard deviation; MVPA, moderate-to-vigorous physical activity; %, percentage; $\dot{\text{V}}$O_2max_, maximal oxygen consumption; HRPF, health-related physical fitness; SBJ, standing broad jump.

* , statistical significance.

Boys significantly (*p* < 0.001) outperformed the girls in all the locomotor abilities (10 m and 20 m speed agility) and the qualitative and quantitative aspects of object control skills for kicking while girls displayed significantly better quantitative balancing skills (37.11 ± 11.28 vs. 33.91 ± 11.60). No significant (*p* > 0.05) sex differences were found in the remaining variables: balance qualitative, catch quantitative, catch qualitative and SBJ quantitative.

Table 4a and 4b highlights the objectively measured PA characteristics of the groups, by sex. In this regard, the analysis of PA behaviour showed significant sex differences (*p* < 0.001) where boys (*n* = 150) participated significantly more in moderate (54.18 ± 17.12) and vigorous (24.10 ± 64.73) PA than girls (45.38 ± 13.35 and 19.93 ± 10.65). Boys also displayed significantly (*p* < 0.001) higher means for total MVPA (71.82 ± 25.21) and average MVPA (78.28 ± 26.89) per day, and 73% of boys met the recommended daily MVPA compared with the girls (58%, *p* < 0.002). Also, boys had a significantly higher mean (793.73 ± 55.09) wear time than girls (781.68 ± 50.95, *p* = 0.05). No significant (*p* > 0.05) sex differences were apparent for sedentary and light PA behaviour.

**TABLE 4a:** Physical activity as measured objectively by using the Actigraph accelerometer.

Objectively measured PA	mean ± s.d.			*p*-value of sex differences
	Group	Males	Females	
Sedentary (min/day)	372.33 ± 58.94	367.69 ± 62.68	377.01 ± 54.97	0.170
Light PA (min/day)	343.57 ± 54.02	347.76 ± 52.60	339.36 ± 399.03	0.180
Moderate PA (min/day)	49.79 ± 15.96	54.18 ± 17.12	45.38 ± 13.35	< 0.001
Vigorous PA (min/day)	22.02 ± 11.68	24.10 ± 64.73	19.93 ± 10.65	< 0.001
MVPA (min per day)	71.82 ± 25.21	78.28 ± 26.89	65.31 ± 21.60	< 0.001
Average wear time	787.73 ± 53.32	793.73 ± 55.09	781.68 ± 50.95	0.050

PA, physical activity; MVPA, moderate-to-vigorous physical activity; s.d., standard deviation; *n*, number; %, percentage.

**TABLE 4b:** Physical activity as measured objectively by using the Actigraph accelerometer.

Variable	Group		Males		Females		*p*-value of sex differences
	*n*	%	*n*	%	*n*	%	
**PA levels using the ActiGraph accelerometer**	-	-	-	-	-	-	0.002
Meeting recommended daily 60 min MVPA per day	197	66	110	73	87	58	-
Not meeting recommended 60 min MVPA per day	102	34	40	27	62	42	-

PA, physical activity; MVPA, moderate-to-vigorous physical activity; s.d., standard deviation; *n*, number; %, percentage.

Table 5 presents the results of both the Spearman correlation coefficient (*r*) between the PA levels and HRPF – and MRPF components and the adjusted partial correlation coefficient (*r*) for sex, BC and SES in determining the associations between the PA levels of the group and their health related – and motor-related fitness and motor skills characteristics. Spearman correlation coefficients revealed a small significant (*p* = 0.05) negative correlation coefficient between sedentary behaviour and the speed tests (10 m, *r* = −0.16 & 20 m run, *r* = −0.13). However, there were small (*p* = 0.05) and moderate (*p* = 0.01) significant negative correlation coefficients, respectively, observed between light (*r* = −0.12; *r* = −0.12), moderate (*r* = −0.13; *r* = −0.16), vigorous (*r*= −0.36; *r* = −0.37), total MVPA (*r*= −0.23; *r* = −0.27) and 10 m and 20 m speed tests. Significant weak negative associations (*r* = −0.12, *p* = 0.05) were found between light activity and the agility fitness item. Moderate vigorous physical activity was weak and negatively (*r* = −0.13, *p* = 0.01) correlated with the quality of kicking. Small to moderate significant positive correlation coefficients were also found between the moderate, vigorous, total MVPA and the MVPA with the beep, $\dot{\text{V}}$O_2_max, agility, qualitative and quantitative aspects of balancing and catching (MRPF) test items.

**TABLE 5:** Correlations (*r*) between physical activity levels and health-related physical fitness – and motor-related physical fitness components.

Physical activity levels	Beep	$\dot{\text{V}}$O_2_max	SBJ	Speed 10 m	Speed 20 m	Running quantitative	Running qualitative phase	Agility	Balance quant	Balance qual	Catch quant	Catch qual	Kicking quant	Kicking qual
Sedentary (min/day)	−0.00	−0.06	0.06	−0.16**	−0.13*	0.10	0.10	0.09	−0.02	0.290**	0.06	0.09	−0.02	0.030
Light (min/day)	−0.08	−0.03	−0.24**	−0.12*	−0.12*	−0.089	−0.09	−0.12**	−0.17**	−0.260**	−0.07	−0.09	0.03	−0.030
Moderate (min/day)	0.19**	0.23**	0.09	−0.13*	−0.16**	0.09	0.02	0.07	0.03	−0.170	0.03	−0.03	0.08	0.090
Vigorous (min/day)	0.37**	0.39**	0.36**	−0.36**	−0.37**	0.16**	0.09	0.29**	0.21**	0.020	0.14*	0.08	0.09	0.030
MVPA (min/day)	0.28**	0.32**	0.22**	−0.23**	−0.27**	0.12**	0.05	0.16**	0.13**	−0.090	0.09	0.02	0.09	0.080
**Partial correlations adjusted for sex, BC and SES**														
Sedentary (min/day)	0.06	−0.08	0.04	−0.12**	−0.08	0.02	0.07	0.05	−0.09	0.170**	0.07	0.05	0.03	0.080
Light (min/day)	0.02	0.09	−0.03	0.07	0.03	−0.04	−0.03	−0.01	−0.08	−0.090**	−0.09	−0.12*	0.05	−0.040
Moderate (min/day)	0.18**	0.23**	0.21**	−0.05	−0.12*	0.08	0.02	0.16*	0.09	−0.007	0.04	0.02	0.03	0.001
Vigorous (min/day)	0.27**	0.30**	0.28**	−0.20**	−0.23**	0.09	0.03	0.30**	0.17**	0.600	0.14*	0.11	0.04	−0.040
MVPA (min/day)	0.24**	0.27**	0.26**	−0.13*	−0.18**	0.09	0.02	0.21**	0.13*	0.011	0.09	0.06	0.02	−0.020

$\dot{\text{V}}$O_2max_, maximal oxygen consumption; quant, quantity; qual, quality; SBJ, standing broad jump; MVPA, moderate-to-vigorous physical activity; BC, body composition; SES, socioeconomic status.

* , *p* = 0.05;

** , *p* = 0.01.

The adjusted analysis of sex, BC and SES in the correlation coefficients between sedentary behaviour and speed tests showed that the strength of the correlation coefficient (*r*) association was significantly (*p* = 0.01) reduced (*r* = −0.16 vs. *r* = −0.12). Furthermore, the adjustment of the sex, BC and SES in the associations between PA levels and HRPF – and MRPF components demonstrated significant reductions in the magnitude of the negative or positive correlation coefficients (*r*) of moderate, vigorous, total MVPA, MVPA with 10 m, 20 m speed tests, beep, $\dot{\text{V}}$O_2_max, agility, balance qualitative and quantitative and catch MRPF test items. After the adjustment of sex, BC and SES the significant correlations between light activity and balance quantity diminished and reduced in the quality of balancing correlation (*r* = −0.09). Also, the adjustment for sex, BC and SES in correlation coefficient (*r*) between sedentary and the quality of balancing skills reduced from *r* = 0.29 to *r* = 0.17. In addition, reductions in the correlations between PA and the quantity and quality of balancing skills after the adjustments were observed.

### Physical activity and health-related physical fitness

After confirming the significant relationship between the PA levels and the health- and motor-related fitness of the group adjusted for sex, BC and SES (Table 5), further analyses were conducted to determine the significance of each of the different role players in the relationship between MVPA and health-related fitness (number of beeps, $\dot{\text{V}}$O_2max_ and SBJ) (see Table 6). The results of a Spearman’s correlation analysis between objectively measured PA and HRPF show a significant, but low correlation between MVPA and the number of beeps (*r* = 0.28) and SBJ (*r* = 0.21), while a moderate correlation between MVPA and $\dot{\text{V}}$O_2_max (*r* = 0.31) was observed (see Table 5).

**TABLE 6:** Spearman correlation (*r*) objectively measured physical activity and health-related physical fitness.

Body fatness, SES and MVPA	Beep level	$\dot{\text{V}}$O_2max_	SBJ
**Zero-order correlation**			
MVPA	0.22*	0.28*	0.32*
**Partial correlations adjusted for sex, BC and SES**			
Controlled for Sex	0.24*	0.28*	0.17*
Body fat%	0.23*	0.26*	0.13*
FFM	0.29*	0.32*	0.26*
SES education	0.29*	0.33*	0.26*
Employment	0.30*	0.33*	0.25*
Income	0.29*	0.32*	0.31*

BC, body composition; SES, socioeconomic status; SBJ, standing broad jump; MVPA, moderate-to-vigorous physical activity; FFM, fat-free mass; $\dot{\text{V}}$O_2max_ = maximal oxygen consumption.

* , *p* < 0.001.

These results show that the positive correlation between the MVPA and the beep levels was still small/weak (*r* = 0.24) after controlling for sex. When controlling for FFM (*r* = 0.29) and SES related to education (*r* = 0.29) employment (*r* = 0.30) and income (*r* = 0.28), the correlation between MVPA and beeps showed significant increases (*p* < 0.001) to a moderate level. However, an inspection of the zero-order correlation (*r* = 0.28) suggests that controlling for sex and BF% has a very small effect on the relationship between the two variables.

A moderate, positive and significant correlation was also established between MVPA and $\dot{\text{V}}$O_2max_ (*r* = 0.28), controlling for sex: *r* = 0.28, BF%: *r* = 0.26 although the magnitude of the correlation decreased substantially. In contrast, controlling for FFM (*r* = 0.32), SES (education: *r* = 0.33, employment: *r* = 0.33 and income: *r* = 0.32), the magnitude of the correlation showed a small increase compared with the zero-order correlation (*r* = 0.28, *p* < 0.001) with an increase in the relationship between MVPA and $\dot{\text{V}}$O_2max_. The zero-order correlation (*r* = 0.28) suggests that adjusting for sex and BF% had little impact on the relationship between the two variables. However, adjusting for FFM, SES education, employment and income resulted in a moderate increase in the zero-order correlation, indicating a moderate impact on the relationship between these variables.

The moderately significant correlation between MVPA and beep levels (*r* = 0.22), increased in magnitude when gender control was applied to *r* = 0.24 and BF% to *r* = 0.24. When controlling for FFM (*r* = 0.29) and SES (education: *r* = 0.29, employment: *r* = 0.30 and income: *r* = 0.29), the magnitude of the correlation; however, significantly (*p* < 0.001) increased compared with the zero-order correlation (*r* = 0.22) revealing an increase in the relationship between MVPA and beep levels.

Furthermore, the results also show a moderate significant correlation (*r* = 0.32) between MVPA and SBJ. When partial correlation analysis was performed between MVPA and SBJ controlling for sex: *r* = 0.17 and BC (BF%: *r* = 0.13), the magnitude of the relationship significantly decreased compared with the zero-order correlation. The zero-order correlation (*r* = 0.21) again suggests that controlling for sex and BF% had very little effect on the relationship between these variables and MVPA. With the inclusion of FFM (*r* = 0.26) and SES (education: *r* = 0.26, employment: *r* = 0.25 and income: *r* = 0.31), the relationship, however, shows significant (*p* < 0.001) increases between MVPA and SBJ.

### Physical activity and motor fitness

The relationship between objectively measured MVPA and MRPF was further explored using a partial correlation analysis (Table 7) while controlling for sex, BC (BF% and FFM) and SES. The results showed a small or weak significant negative relationship between the MVPA and 10 m speed run (*r* = −0.24) and a small or weak significant positive relationship between the MVPA and the quality of running (*r* = 0.12). Once these factors were considered, a small or weak significant (*p* < 0.001) correlation between speed across 10 m and 20 m and agility was found.

**TABLE 7:** Correlation (*r*) between objectively measured physical activity and selected motor fitness and motor skills.

Body fatness, SES and MVPA	Speed 10 m	Speed 20 m	Running qualitative	Running quantitative	Agility	Balance quant	Balance qual	Catch quant	Catch qual	Kicking quant	Kicking qual
**Zero-order correlation**											
MVPA	−0.24*	−0.27*	0.12*	0.05	0.22*	0.07	0.13*	0.09	0.02	0.09	0.07
**Partial correlations adjusted for sex, BC and SES**											
Sex	−0.12*	−0.17*	0.10	0.02	0.17*	0.10	0.15*	0.04	−0.00	0.03	0.00
BF%	−0.11*	−0.17*	0.09	0.03	0.13*	0.06	0.07	0.08	0.02	0.07	0.04
FFM	−0.21*	−0.27*	0.13*	0.06	0.26*	0.07	0.11	0.12*	0.05	0.09	0.10
SES, Education	−0.18*	−0.24*	0.12*	0.05	0.26*	0.08	0.12*	0.07	0.01	0.07	0.08
Employment	−0.18*	−0.24*	0.12*	0.05	0.25*	0.08	0.12*	0.07	0.02	0.07	0.08
Income	−0.19*	−0.24*	0.16*	0.09	0.30*	0.10	0.14*	0.11	0.05	0.07	0.08

Note: 0.0 and 0.3 = a small or weak correlation; and values of 0.30–0.50 indicate a medium or moderate correlation and ≥ 0.50 indicates a larger or strong correlation.

BC, body composition; SES, socioeconomic status; SBJ, standing broad jump; MVPA, moderate-to-vigorous physical activity; FFM, fat-free mass; BF, body fat; quant, quantity; qual, quality.

* , *p* < 0.05.

However, the zero-order correlation shows that after correcting for sex, BC and SES (*r* = −0.24; *r* = −0.27; *r* = 0.22), sex and BF% had only a very minor effect on the correlation between the two variables (speed 10 m: *r* = −0.12 and *r* = −0.11; speed 20 m: *r* = −0.17 and *r* = −0.17; agility: *r* = 0.17 and *r* = 0.13). However, all the SES factors and FFM showed a moderate small or weak negative significant correlation between speed across 10 m and 20 m and agility. It also showed a small or weak positive correlation between the MVPA and running quality. The zero-order-correlation shows that correcting the FFM (*r* = 0.13) and SES factors of education (*r* = 0.12), employment (*r* = 0.12) and income (*r* = 0.16) had little effect.

Overall, the results show that all SES characteristics and FFM slightly correlated with MRPF, but sex and BF% had no significant influence on the correlations.

### Physical activity and motor skills

There was a small or weak and positive correlation between the MVPA and the quality of balancing when controlling for sex: *r* = 0.15 (*n* = 29, *p* < 0.001), SES (employment: *r* = 0.12 and income: *r* = 0.14). Furthermore, an inspection of the zero-order correlation (*r* = 0.126) also indicates that controlling for sex, BF% and FFM had a very small effect on the relationship between these two variables.

The partial correlation between MVPA and kicking accuracy (*p* = 0.155) and catching accuracy (0.923) was not statistically significant (*p* > 0.05), suggesting that even when controlling for sex, BF% and FFM, this did not affect the correlation between the variables of interest.

## Discussion

This study examined the influence of sex, SES factors (education level, employment status and income, and BC (underweight, overweight and obesity) on the relationship between PA, specifically MVPA, HRPF, MRPF and motor skills in children with a mean age of 6.83 years. Several novel findings were derived from this study. Firstly, body fat percentage (BF%) and sex did not influence the associations between MVPA, HRPF and MRPF. Secondly, FFM and SES factors had small to moderate effects on the association between HRPF, MRPF and MVPA. However, in the case of adjustments to FFM and SES for education, employment and income, the zero-order correlation indicated a moderate influence on the relationship between these two variables. Thirdly, sex, BC and SES do not have statistical correlations with any of the motor skills in the MVPA relationship, except for balancing abilities that showed a small favourable relationship with the MVPA.

The group revealed acceptable PA levels although higher activity rates were expected for such young children. In the group, 66% met MVPA recommendations for 60 min of the MVPA per day, with significantly (*p* < 0.02) high percentage in boys (73%) compared with girls (58%) (Table 4a and 4b). In terms of the sex differences concerning who meets the recommended MVPA per day, findings in our study are slightly lower than percentages reported by a recent study in a peri-urban area in the Eastern Cape of South Africa with 91.0% in boys and 62.4% in girls.^5^ The difference observed in the two studies (i.e. Eastern Cape vs. North West province) can be explained by the differences in the distance children had to commute between schools and residential areas. The majority of children from the Eastern Cape’s study were from rural areas (*n* = 300; 65.4%) versus North West province (*n* = 299; urban and township), where most learners tend to walk long distances and engage in more physical household chores compared with those in the urban and peri-urban settings.^48^ In addition, the International Children’s Accelerometery Database revealed that objectively measured PA (although based on cross-sectional data) may be in decline in both sexes from around the age of school entry.^49^ A longitudinal study that measured PA across childhood and adolescence in children born in Iowa in the early 1990s also reported that PA declined in both sexes, from age 5 years in most of the study participants.^50^ It is also aligned with the boy-girl differences in the MVPA levels that are reported by studies of younger children, showing lower PA levels in girls. In this regard, girls are more likely than boys to experience the PA impacts at the school and family levels, as well as through not engaging in extracurricular sports. Furthermore, girls exhibit less favourable BC and fitness characteristics linked to PA, such as lower cardio-respiratory fitness, MC and higher percentages of BF.^51^

Poor BC characteristics were also observed in the sample evaluated, whereby 11% and 8% (i.e. 19% in total) of the participants were classified as overweight and obese, respectively. Compared to boys (3% and 7%), girls were more likely to be overweight 15% and obese 13%. These findings are consistent with the world trends of poorer BC underscored by a larger proportion of individuals being classified as overweight/obese, especially during early and middle childhood.^1,30,37^ In addition, it was reported that low- and middle-income countries are still experiencing a rapid incline in childhood obesity, which led to an increase in inequalities between wealthy and poorer countries as trends show that children residing in poorer circumstances are more likely to be linked to overweight or obesity.^52^ It was also reported that urbanisation and changing lifestyles increase sex differences in obesity prevalence.^5^ Low MVPA is linked to higher obesity rates, particularly after the age of seven, in various studies.^53^ Although 66% of the cohort evaluated in this study met the recommended PA levels per day, 34% still did not comply with the recommended 60 min per day of MVPA, with girls being non-compliant compared with the boys. A better understanding of factors contributing to these unhealthy activity behaviours and BC is needed, especially in young children in the age group between five and eight where activity habits are formed and are often based on good PA and fitness as well as motor skills foundations that can address sedentary behaviours and unhealthy weight.

Our results corroborate the associations between MVPA, HRPF, MRPF and demographic factors associated with SES. More specifically, evidence for an association between parental SES (e.g. education, employment income) and HRPF (*r* = 0.250–0.334) as well as between SES and MRPF (*r* = 0.123–0.305) was weak-to-moderate in 5- to 8-year-old children and are consistent with the findings of other studies.^26,54^ Aerobic fitness, as measured by the 20-m shuttle run and predicted $\dot{\text{V}}$O_2max_ and strength correlated with moderate, vigorous and MVPA levels, and a small significant association was also established between MVPA, FFM and all the assessed SES factors in aerobic fitness. These relations increased to a moderate association with strength as measured by the SBJ although the confounders did not influence the zero-order correlation of strength. The higher level of MVPA that were associated with higher CRF is consistent with the findings of other studies focused on similarly aged children.^5,6^ According to Raghuveer,^54^ the most influential determinants of aerobic fitness in children are age, sex and engagement in regular vigorous PA. Moreover, muscle fitness also plays a relevant role in PA, especially in vigorous-intensity activity.^55^ The study of Gu^56^ in girls aged seven to 10 found that MVPA was positively associated with a higher skeletal muscle mass index and standing long jump distance.

Evidence of studies on parental influence shows that parents’ SES may affect their children’s physical fitness.^25,57^ A study by Merino-De Haro^57^ assessed physical fitness in the 20-m shuttle run and the 4 m × 10 m shuttle run and found pre-schoolers with high paternal occupation had higher speed or agility (*p* = 0.005), and those with high or low maternal education had higher $\dot{\text{V}}$O_2_max (*p* = 0.046). In agreement, studies reported that children between the ages 6 to 11 years with educated parents may have differences in CRF, higher strength in the lower limbs, higher balance, endurance sprint velocity and flexibility.^26^ Higher parental education may be associated with greater participation in sports and recreational activities of children and the increased awareness of PA’s positive health benefits.^58^ It is also reported by Ferreira^58^ that the consistent positive correlates with PA can be the result of the example established by the father’s PA, time spent outdoors and school PA-related policies (in children), support from significant others, the level of mother’s education, family income and non-vocational school attendance, specifically in adolescents. Furthermore, the need for indoor and outdoor physical activities environments and parents encouraging their children to participate actively play an essential role in the organisation of PA.^29^ It should also be observed that the availability of organised sports in cities and rural areas may differ and ultimately may influence PA behaviour.^59^ It is also reported that lower SES home environments provide more opportunities for sedentary behaviour and lower opportunities for PA.^60^

This study also revealed associations between PA levels, MRPF and motor skills, especially with the vigorous and MVPA levels. There were significant associations between vigorous PA and MVPA, with higher performance in MRPF (*r* = −0.36). Speed and agility were both correlated with vigorous and MVPA levels, but only agility was strongly influenced by all three SES factors and FFM. Furthermore, the results showed significant associations between MVPA and motor skills (*r* = 0.13) specifically with the qualitative and quantitative aspects of balancing, while catching was only associated with vigorous activity (*r* = 0.14). Different motor skills may require different levels of physical exertion and may therefore be closely associated with activities that involve greater intensity or duration. Parental income played an important role in adjusting the analyses of associations in both skills. Previous studies also reported positive associations between PA and the motor skills of children aged between 3 and 9 years.^22,61^ According to Wrotniak,^22^ more active children complete the tasks of speed and agility faster and they were able to jump further. This suggests that running speed and agility and SBJ may be important to consider while studying the MC-PA relationship or while trying to improve MC in youth. These movement skills can be fundamental tasks to promote stronger and longer endurance for active games and sports participation. Wrotniak^22^ also found a significant association between the BOTMP (which evaluates multiple fitness and motor components), standard scores, activity counts per minute and percentage of time in sedentary PA (*r* = −0.31; *p* = 0.012), moderate PA (*r* = 0.33; *p* = 0.008) and MVPA (*r* = 0.30; *p* = 0.016).

In summary, the findings of this study firstly confirm the literature that indicates the relationship between MVPA, health and motor-related fitness and motor skills at a young age, and secondly, that it is influenced by factors associated with SES (educational level, employment status and income) and FFM although to different degrees in each of these areas of fitness competence. Further research should include information on potential barriers or challenges preventing children from participating in regular PA and provide strategies to overcome these obstacles. In this regard, initiatives are needed to increase access to safe and affordable PA options in low-income neighbourhoods. Educational programmes can also be developed and be implemented by volunteers and youth in such communities in helping children and more specifically parents to understand the importance of PA and healthy eating and to further provide them with the necessary resources they need to support their children’s health.

The strengths of this study centre on the analyses of the various influences of BC and SES on PA levels, BC, HRPF, MRPF and motor skills and especially the use of objectively measured PA and BIA for determining the levels of overweight and obesity. Additionally, the relationship between MVPA and motor skills was assessed at both qualitative and quantitative levels, making this a unique contribution of this study. Another strength is that the study was conducted in children aged five to eight, an age that is important for the development of positive exercise behaviours that may have a long-term impact on health and well-being. Few studies have been reported in this age group, especially in South African children, which strengthened the understanding of the associations that need attention at this age created by this study’s findings. However, this study also had limitations that must be recognised. Based on cross-sectional data and a sub-sample from the ExAMIN-Youth study, these data are neither representative of the larger population it represents nor of the whole of South Africa. Therefore, the generalisability of the results is limited, and extrapolation to the larger population of children should be interpreted with caution. Cross-sectional data cannot also be used to test the cause-and-effect relationships; therefore we recommend longitudinal research to address the challenges highlighted in this study to reduce physical inactivity and the prevalence of childhood obesity.

## Conclusion

The findings inferred that MVPA is moderately associated with HRPF and MRPF but is less associated with all motor skills. It is, therefore, necessary to recognise the direct influences of socioeconomic factors such as education, income and parental employment on MVPA and to identify strategies to address these challenges at an early age. Healthy BC (FFM) is also associated with improved HRPF, MRPF and MVPA. One-third of the group did not meet the recommended level of MVPA, and obesity, especially in girls, was widespread, highlighting the importance of identifying and addressing factors that contribute to these unhealthy habits and conditions in early childhood. Boys have a higher level of MVPA and better movement and object control skills than girls. However, more research is needed to better understand the relationship between MVPA, motor skills and SES, and to design targeted interventions to promote PA and healthy habits among children, especially those hampered by the characteristics of poor SES.
